# Community Violence and Associated Psychological Problems Among Adolescents in the General Population

**DOI:** 10.1007/s40653-018-0218-8

**Published:** 2018-06-02

**Authors:** Christine Dubé, Marie-Hélène Gagné, Marie-Ève Clément, Claire Chamberland

**Affiliations:** 1 School of Psychology, Université Laval, 2325 rue des Bibliothèques, Pavillon Félix-Antoine-Savard 11ème étage, Québec, Québec G1V 0A6 Canada; 2 Present Address: Centre Hospitalier de l’Université Laval, Québec City, Canada; 3 Psychology and Psychoeducation Department, Université du Québec en Outaouais, Saint-Jérôme, Canada; 4 School of Social Work, Université de Montréal, Montréal, Canada

**Keywords:** Context of violence, Populational survey, Trauma, Anger, Depression, Physical violence

## Abstract

Based on a populational survey conducted among 1400 adolescents aged between 12 and 17 years old, the aim of this study is to assess the relationships between their community violence experiences and their psychological health (anger, depressive symptoms, and post-traumatic stress disorder symptoms). One MANOVA confirms that both boys and girls who report at least one incident of physical community violence present more psychological difficulties, especially anger. Subsequent MANOVAs show that anger intensity varies depending on whether the youth was a direct victim or a witness only, as well as on the diversity of the types of violent manifestations and on acquaintance with the perpetrator, whereas the presence of injuries has no significant effect. This study highlights the importance of considering the context of the community violence incident, to clearly understand its relationships with the youth’s psychological difficulties.

Mental health problems affect 10–20% of children and adolescents worldwide (Kieling et al. 2011). To illustrate, a survey carried out in Quebec, Canada (Institut de la statistique du Québec - ISQ 2013) reveals that one out of five adolescents presents a high level of psychological distress. Such distress is associated with the level of adversity that characterizes youth’s environment, notably being in contact with violent or unsupportive others (ISQ 2013). In adolescence, young people gradually access a greater variety of environments, which exposes them to new forms of adversity such as community violence (Matjasko et al. 2013). Focusing on adolescents from the general population in a high-income country (Canada), the present study evaluates whether youth who have experienced physical community violence differ from those who have not on various indicators of psychological health, and which characteristics of the violent incident can account for various symptoms.

According to the World Health Organization’s conceptual framework, community violence is a form of interpersonal violence perpetrated by strangers or acquaintances other than family members or intimate partners (Krug et al. 2002). Available data suggest that community violence is a widespread social issue. According to a 2011 US victimization survey (Finkelhor et al. 2013), 21.1% of 10–13-year-old and 36.4% of 14–17-year-old adolescents witnessed an assault in their community during last year. Another study performed with a large sample of 9–18-year-old youths from seven European countries revealed that 76% of youth diagnosed with conduct problems reported at least one incident of community violence during last year, as compared to 34% of those without such diagnosis (52% overall) (Kersten et al. 2016). In Québec, Canada, a survey representative of 12–17-year-old adolescents showed that 35.2% of participants reported experiencing at least one form of community violence in the past year, as a victim or a witness (Dubé et al. 2014).

A literature review suggested that being a victim or witness of community violence is a predictor of psychological distress in urban youth, especially depressive symptoms, anxiety, post-traumatic stress (PTS), and aggression (McDonald and Richmond 2008). Enlarging the focus to all youths aged 25 or less, Fowler et al. (2009) summarized the empirical literature available up to July 2005 in a meta-analysis of 114 published and unpublished studies linking community violence to three categories of psychological symptoms: externalized behaviors, internalized behaviors, and PTS. Overall, their findings confirm strong associations between community violence and PTS symptoms (*d* = .786) as well as externalized behaviors – especially aggressive behavior (*d* = .630), and moderate association with internalized behaviors (*d* = .454). These findings are supported by recent research in various countries (e.g.: Bacchini et al. 2011; Nöthling et al. 2016; Yearwood et al. 2017).

To understand more deeply the relationship between community violence and psychological problems in adolescents, it is important to consider moderating variables. McDonald and Richmond (2008) reviewed the following moderators: sex of the victim, family relationship characteristics, school connectedness, parental mental health, ethnicity, and grade level. Overall, findings are inconsistent regarding the moderating role of these variables. In addition to adolescent’s sex, the present study focuses on the characteristics of community violence incidents as potential moderators of the relationship between victimization on one hand, and symptomatology on the other hand (angry, depressive, and PTS symptoms). These characteristics are: victimization repetition, proximity (victim vs. witness), perpetrator’s identity, and severity (presence of injury).

## Sex

Generally, boys report more community violence than girls (Rose and Rudolph 2006), except for sexual violence which is more commonly reported by girls (Tolin and Foa 2006). However, the sex of the victim does not appear to alter the relationship between community violence and psychological symptoms (McDonald and Richmond 2008). This hypothesis will be tested in the present study.

## Multiple Victimization

Polyvictimization research has shown that any experience of victimization is a predictor for further similar experiences (Saunders 2003; Turner et al. 2010). Youth who are victimized a first time are two to seven times more likely to be revictimized within a year in a similar or different context, compared with those who are not victimized (Finkelhor et al. 2007). In certain contexts, young victims and witnesses tend to experience violent events regularly, even daily (Foster et al. 2004), and being a frequent victim or witness of community violence has been associated with more psychological problems (Scarpa et al. 2006).

## Victim Versus Witness

As it relates to being a victim or a witness, research results prove contradictory. According to some authors, being a direct victim of a violent event rather than only a witness is associated with greater symptomatology (Lynch 2003), while others found no significant difference in reported symptoms between victims and witnesses (Kennedy and Ceballo 2014). According to Zona and Milan (2011), being the victim of a violent event predicts aggressive and depressive symptoms, whereas being a victim or a witness is a predictor of PTS symptoms. This finding reflects the Fowler et al. (2009) meta-analysis: even though direct victimization most predicted symptomatology compared to witnessing community violence, PTS symptoms were equally predicted by victimization or witnessing.

## Perpetrator’s Identity

Another variable that may explain the level of psychological difficulties in young victims of community violence is the relationship between the victim and the perpetrator; that is, whether the victim is acquainted or not with the perpetrator. Interpersonal traumas are more strongly correlated with psychological distress and mental health problems than non-interpersonal traumas (Price et al. 2013). When the perpetrator is an acquaintance, the effects are more deleterious than when the perpetrator is a stranger, given the attachment issues and the relationship of trust initially established (Kennedy and Ceballo 2014; Lynch 2003). Therefore, being the victim of community violence by someone close has been associated with more depressive and PTS symptoms as well as more aggressive behaviors than being victimized by a stranger (Lambert et al. 2012; Kennedy and Ceballo 2014).

## Injuries

Violence involving injuries, especially those severe enough to damage the victim’s physical integrity, has been associated with more PTS symptoms. A violent event involving bodily harm increases the risk of developing PTS by more than eight times (Kennedy and Ceballo 2014; Martin et al. 2006). However, it seems that no study has examined how the presence of injuries resulting from community violence is related to depressive or aggressive symptoms in the victims. This will be explored in the present study.

This body of empirical literature has some limitations. Several studies use samples of high-risk children or adolescents, often boys only, living in disadvantaged urban neighborhoods, of low socio-economic status or consisting solely of African Americans, in the United States (Scarpa 2003). These populations are typically affected by several other risk factors, such as poverty, inadequate health care or overpopulation (Lynch 2003). These confounding variables could thus explain part of the psychological problems linked to community violence. This study overcomes these gaps by examining community violence among both male and female adolescents, from the general population. It uses a large adolescent sample recruited in the context of a population survey (Cyr et al. 2013). It focuses on physical assaults, since 31.0% of respondents reported being victim and/or witness of such assaults in their community. In comparison, 4.8% reported living in a climate of violence (e.g., hate crimes, intimidation, hearing about violence in the community…), and 5.1% reported community sexual violence (from exhibitionism to rape) (Dubé et al. 2014).

## Objectives and Hypotheses

The purpose of this study is to document the relationships between community violence exposure and three indicators of psychological health in youth. It is expected that (1) youth reporting a physical community violence experience in the year preceding the survey will present more anger, PTS symptoms, and depressive symptoms; and (2) there will be no interaction effect between sex and violence exposure to explain psychological symptoms.

Among youth reporting at least one experience of physical community violence during the year, the study aims to evaluate whether multiple victimization experiences, as well as being a victim rather than only a witness, leads to differences in youth’s psychological health. On this subject, it is expected that (3) youth reporting various manifestations of physical community violence will report more anger, PTS symptoms and depressive symptoms than youth who report only one manifestation; and (4) youth who were victims of physical assault in their community will report more anger than those who were only witnesses of such assaults.

Finally, among young victims, the study aims to document whether being acquainted with the perpetrator and sustaining injuries lead to differences in the psychological health of youth having suffered physical community violence. In this regard, it is expected that (5) youth who were acquainted with their perpetrator will present more anger, PTS symptoms, and depressive symptoms than those assaulted by a stranger; and (6) youth who were physically injured will present more PTS symptoms than those who were not.

## Method

### Sample

This study analyses data collected during a survey on polyvictimization of Québec adolescents between 12 and 17 years of age (Cyr et al. 2013), based on the methodology developed by Finkelhor et al. (2005). A telephone survey on the theme of polyvictimization was conducted in 2009 among 1400 Québec adolescents (49.7% boys), in French and in English. More than 85% of them were Caucasian. The base for this survey was a list of phone numbers created using a random generation technique (Kish 1965). Despite the sample being random, the respondents came from families that were slightly more educated than the general population (Nobert 2009). In this study, 72% of the youth’s parents had completed postsecondary studies, compared with 60% in Québec’s general population (Cyr et al. 2013).

The response rate was 37.6%, which is acceptable according to current survey standards (Babbie 2007). Indeed, studies tend to show that low response rates in telephone surveys do not seem to influence the representativeness of the data. Studies having attempted to maximize their response rates obtained only a small effect on data representativeness (Holbrook et al. 2007).

### Measures – Victimization Variables

#### Juvenile Victimization Questionnaire

The *Juvenile Victimization Questionnaire* (JVQ – Hamby et al. 2005) was the source for all the victimization variables, as it measures 34 different manifestations of violence (sustained or witnessed), grouped into five categories: conventional crimes, child maltreatment, peer and sibling victimization, sexual violence, and witnessing / indirect victimization. For each of these manifestations, the JVQ documents lifetime and last-year frequency, presence and nature of physical injuries, as well as the number of perpetrators, their sex, and their identity.

#### Experience of Physical Community Violence

The community violence measure derived from the JVC by Dubé et al. (2014) is used in this study to measure community violence exposure during the last year. These authors performed an exploratory factorial analysis on 27 JVQ violence manifestations, perpetrated by peers or adults other than family members or intimate partners, that matched WHO’s definition of community violence. The resulting measure includes a scale of physical community violence, comprising the six following manifestations: victim of simple assault, witness of simple assault, victim of armed assault, witness of armed assault, assault by a group, and attempted assault. For the purposes of this study, a youth reporting at least one occurrence on at least one of these items, involving a non-related perpetrator (peer or adult) was considered to have experienced physical community violence and was coded (1); others were coded (0).

In order to account for some characteristics of physical community violence experience, the following variables were created from JVC answers: (1) diversity of manifestations of physical community violence (one vs. more than one); (2) having been a victim or only a witness of physical community violence; (3) having been the victim of an acquaintance vs strangers only; and (4) having sustained (or not) injuries related to this violence.

#### Diversity of the Manifestations of Physical Community Violence

This variable was calculated for youth reporting at least one experience of physical community violence. From the six manifestations of physical violence measured, one dichotomous variable was created based on whether the youth reported only one type of manifestation (0) or more than one type of manifestation (1).

#### Witness or Victim

This variable was also calculated for youth having experienced physical community violence. Among the six manifestations measured, some refer to direct victimization, while others describe indirect victimization (being only a witness to an incident of violence). A dichotomous variable was created to distinguish between these two types of experiences. Youth reporting at least one manifestation of direct victimization are coded (1), whether or not they were also a witness of violence. Those who reported being only a witness of violence are coded (0).

#### Victim of Stranger Vs. Acquaintance

This variable was calculated for youth who were direct victims of violence. It was created from a JVQ sub-question that documents perpetrator’s identity. For all manifestations of physical community violence reported, if the perpetrator was an acquaintance in at least one case, the youth is coded (1). Youths who report being victims of strangers only are coded (0).

#### Presence of Injuries

This variable was also calculated for youth who were direct victims. It was created based on a sub-question from the JVQ that asks whether any injuries, ranging from bruises to loss of consciousness, resulted from the violent event. As part of this study, youths who reported never being injured for any of the manifestations of physical community violence were coded (0). Youths who reported being injured at least once for at least one manifestation of physical violence were coded (1), regardless of the nature of the injuries reported.

### Measures – Psychological Variables

#### Trauma Symptom Checklist for Children

The *Trauma Symptom Checklist for Children* (TSCC) developed by Briere et al. (1995) is a questionnaire intended for 8- to 16-year-old boys and girls who have been victims or witnesses of traumatic events. It measures various feelings, thoughts and behaviors experienced or exhibited in the previous month. The French translation of the TSCC (Wright and Sabourin 1996) has been validated by Jouvin (2001) in normative and clinical samples. Three of the six scales from the instrument are used in this study. The Depression scale (9 items, α = .84) measures feelings of sadness and loneliness, accompanied by episodes of crying, feeling guilty, pessimism, or suicidal thoughts. The Anger scale (9 items, α = .81) measures angry or hateful thoughts, the desire to scream, to fight, and to insult others, as well as nervousness. The PTS scale (10 items, α = .84) measures the youth’s preoccupation with recent or past traumatic events, accompanied by overwhelming thoughts that could lead to irritability, distraction or tension (Jouvin 2001). Each item is measured according to a four-point frequency scale (“never” to “almost always”). A score for each scale is calculated by adding up the answers to each item. In this sample, the internal consistency is α = .63 (depressive symptoms), α = .73 (anger) and α = .78 (PTS).

### Procedure

Data were collected through computer-assisted telephone interviews, conducted by a firm specializing in large-scale social surveys. When used to address sensitive subjects, telephone surveys generate results comparable or superior to in-person interviews. Because this type of survey preserves the anonymity of the respondents, they are more comfortable and tend to respond more conscientiously (Reddy et al. 2006).

When making the calls, the interviewer would first verify whether the household included at least one 12- to 17-year-old youth who was inclined to participate in the study. Next, sociodemographic information on the family was obtained. Finally, the interviewer would make sure to receive verbal consent from youth aged 14 years or over, or from the parents of children under 14 years old, as applicable. Ethical review boards of the researchers’ universities approved the survey.

### Analysis Strategy

First, descriptive analyses were performed on the victimization variables (*n* and %) and on the psychological variables (*M* and *SD*). Next, three bivariate correlation matrices (*r*) were used to document the relationships between the independent variables (victimization variables and youth’s sex) and the dependent variables (psychological variables).

To meet the study objectives and to verify the hypotheses, a series of 2 × 2 factorial multivariate analyses of variance (MANOVAs) was performed. This type of analysis helps to maximize the probability of detecting significant differences between groups by considering the variance shared by the dependent variables and of identifying the interaction effects between the independent variables.

For each significant MANOVA, an a posteriori stepdown analysis was performed using the Roy-Bargmann method (Finch 2007). This procedure consists in adding the different psychological variables, one by one, in order of their theoretical importance, and then removing each variable previously entered in the model while converting it to a covariable. When the dependent variables are correlated, this procedure can be used to detect those that most differentiate the groups under study. In the current case, and based on the literature review, anger was entered first, followed by PTS symptoms (anger withdrawn and then placed as a covariable), and then depressive symptoms (withdrawal of PTS symptoms, which are placed as a covariable).

## Results

Of the 1400 youth in the sample, 435 (31.1%) experienced physical community violence in the year preceding the survey. Among them, 217 (15.5%) reported at least one experience of direct victimization. Youth in the entire sample (*N* = 1400) present mean scores of 5.07 (*SD* = 3.43) for anger, 6.10 (*SD* = 3.78) for PTS symptoms, and 4.29 (*SD* = 2.64) for depressive symptoms. Among adolescents reporting at least one experience of physical community violence (*n* = 435), mean scores were 5.68 for anger, 6.67 for PTS symptoms, and 4.51 for depressive symptoms. Among the direct victims (*n* = 217), mean scores were 6.45 for anger, 7.11 for PTS symptoms, and 4.69 for depressive symptoms.

Correlations were calculated for the different variables of interest based on the sub-samples identified above, namely all the youth in the sample (*N* = 1400), victims and witnesses of physical community violence (*n* = 435), and direct victims (*n* = 217).^1^ Overall, correlations are weak, even though most of them are statistically significant. Moderate to high correlations (.45 to .67) were found between the three psychological variables.

### Main Analyses

A first 2 (experience of community violence) × 2 (sex) factorial MANOVA was performed for the entire sample (*N* = 1400) using the anger, PTS, and depression scores as dependent variables. The results, which are reported in Table 1, show a small and significant main effect of community violence experiences, F (3, 1396) = 7.36, η2 = .02. The stepdown analysis performed according to the Roy-Bargmann method shows that this effect is significant for anger only. Therefore, youth having experienced community violence report significantly more anger than those not reporting such an experience, regardless of their sex. Given the absence of a sex effect and the weak or nil correlations between sex and the other variables under study, sex was not considered in subsequent analyses.

**Table 1:** Differences between youth reporting an experience of physical community violence and thosennot reporting any: MANOVA and stepdown analyses (*N* = 1400)

	Experience of physical community violence				Sex				Experience of physical community violence * Sex							
	Absence (*n* = 965)		Presence (*n* = 435)		Male (*n* = 696)		Female (*n* = 704)		Absence * Male (*n* = 430)		Presence * Male (*n* = 266)		Absence * Female (*n* = 535)	Presence * Female (*n* = 169)		
Variables	*M*	*S.D.*	*M*	*S.D.*	*M*	*S.D.*	*M*	*S.D.*	*M*	*S.D.*	*M*	*S.D.*	*M*	*S.D.*	*M*	*S.D.*
Anger	4.77	3.11	5.62	3.57	5.19	3.40	4.95	3.46	4.79	3.17	5.79	3.62	4.77	3.43	5.39	3.49
PTSD symptoms	5.79	3.56	6.61	4.10	6.30	3.74	5.86	3.80	6.08	3.48	6.62	4.08	5.54	3.60	6.60	4.13
Depressive symptoms	4.18	2.57	4.50	2.77	4.39	2.70	4.20	2.58	4.31	2.63	4.49	2.81	4.07	2.51	4.51	2.71
*F* (3, 1396) (η^2^)	7.36**(.02)				.66 (.00)				1.98 (.00)							
					Stepdown analyses											
Anger *F* (3, 1396) (η^2^)	17.86** (.01)				1.19 (.00)				1.01 (.00)							
PTSD symptoms *F* (4, 488) (η^2^)	2.99 (.00)				.72 (.00)				3.67 (.00)							
Depressive symptoms *F* (5, 487) (η^2^)	1.20 (.00**)**				.07 (.00)				.25 (.00)							

*M* mean, *S.D.* standard deviation, *η*^*2*^ partial eta squared

** *p* < .01 * *p* < .05

A second 2 (diversity of the manifestations) × 2 (witness vs. victim) factorial MANOVA was performed for the 435 adolescents reporting an experience of physical community violence, with the same three dependent variables. The results, which are reported in Table 2, show a small and significant main effect of being a direct victim of physical violence, F (3, 482) = 4.04, η2 = .03. The stepdown analysis shows that this effect is significant for anger only. Direct victims thus report significantly more anger than witnesses only, whether they report only one or several types of manifestations of community violence.

**Table 2:** Differences according to the diversity of the manifestations of violence and being a witness or a victim: MANOVA and stepdown analyses (*n* = 435)

	Diversity of the manifestations of physical violence				Witness or victim of physical violence				Diversity * Witness or victim							
	Only one type (*n* = 313)		More than one type (*n* = 122)		Witness (*n* = 218)		Victim (*n* = 217)		One * Witness (*n* = 199)		More than one* Witness (*n* = 19)		One * Victim (*n* = 114)		More than one * Victim (*n* = 103)	
Variables	*M*	*S.D.*	*M*	*S.D.*	*M*	*S.D.*	*M*	*S.D.*	*M*	*S.D.*	*M*	*S.D.*	*M*	*S.D.*	*M*	*S.D.*
Anger	5.62	3.60	5.84	3.77	4.92	3.12	6.45	3.97	4.94	3.12	4.74	3.16	6.81	4.06	6.05	3.86
PTSD symptoms	6.47	3.95	7.20	4.53	6.25	3.98	7.11	4.25	6.18	3.85	7.05	5.24	6.99	4.10	7.23	4.42
Depressive symptoms	4.47	2.71	4.61	3.09	4.32	2.56	4.69	3.05	4.32	2.48	4.36	3.37	4.73	3.06	4.65	3.05
*F* (3, 482) (η^2^)	1.74 (.01)				4.04** (.03)				.17 (.00)							
					Stepdown analyses											
Anger *F* (3, 482) (η^2^)	.95 (.00)				10.41** (.02)				.32 (.00)							
PTSD symptoms *F* (4, 481) (η^2^)	3.81 (.01)				1.69 (.00)				.08 (.00)							
Depressive symptoms *F* (5, 480) (η^2^)	.45 (.00)				.02 (.00)				.13 (.00)							

*M* mean, *S.D.* standard deviation, + plus, η partial eta squared.

* *p* < .05 ** *p* < .01

**Fig. 1:**
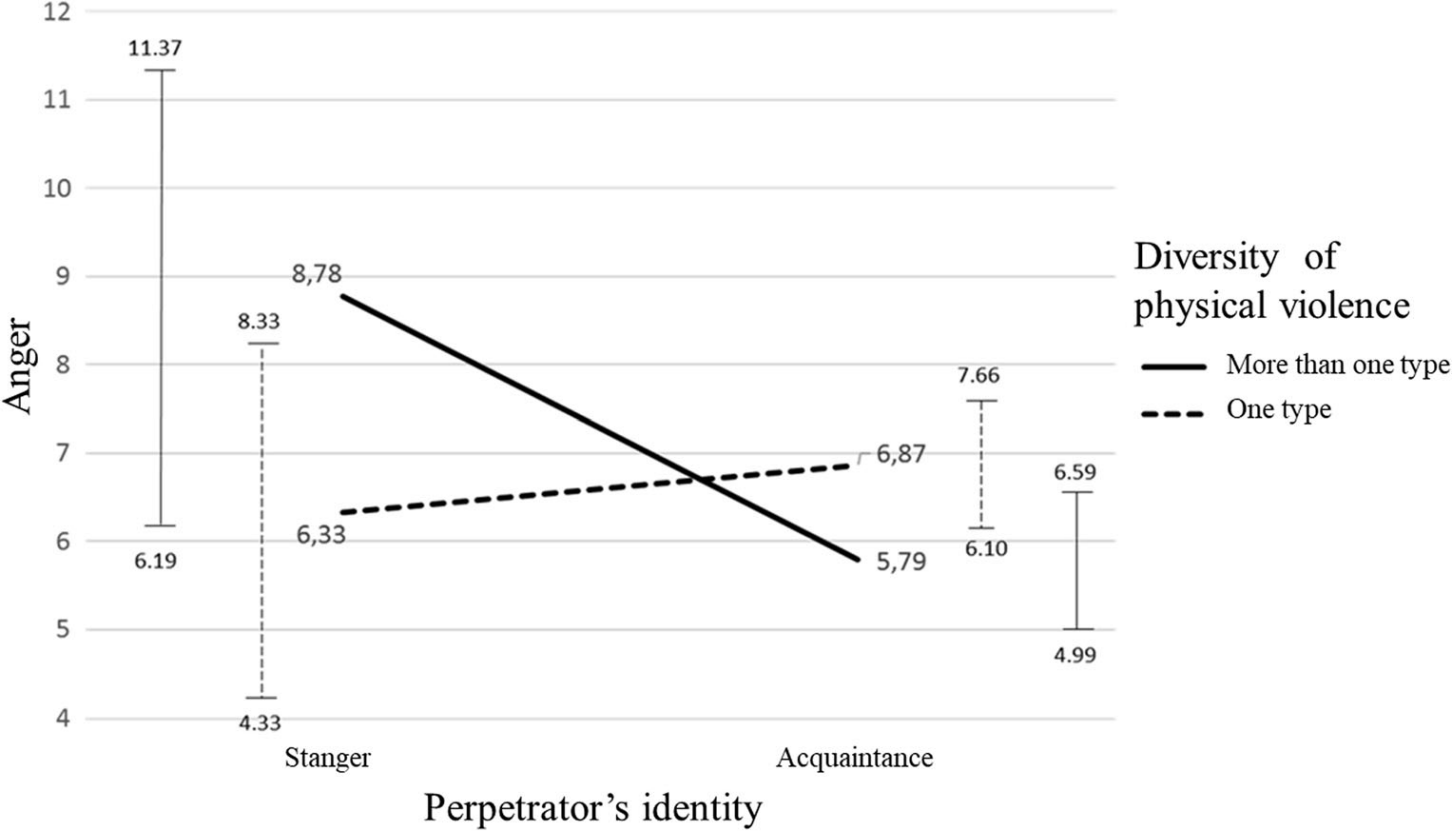
Interaction effect between diversity of violence and perpetrator’s identity on anger (*n* = 217)

A third 2 (diversity of the manifestations) × 2 (acquainted perpetrator vs. stranger) factorial MANOVA was performed for the 217 youth who reported being direct victims of physical community violence, always with the same three dependent variables. The results are reported in Table 3. They show a small effect of the diversity of manifestations of physical community violence, F (3, 207) = 2.68, η2 = .04, characterized by a significant diversity X perpetrator’s identity interaction effect, F (3, 207) = 3.46, η2 = .05. The stepdown analysis shows that this interaction effect, illustrated in Fig. 1, is significant for anger only. Consequently, when the perpetrator is a stranger, anger is significantly higher in youth having suffered diverse manifestations of community violence. This trend is reversed when the perpetrator is an acquaintance; however, in this case, the difference in the mean anger score is not significant.

**Table 3:** Differences according to the diversity of the manifestations of violence and the perpetrator’s identity: MANOVA and stepdown analyses (*n* = 217)

	Diversity of the manifestations of physical violence				Perpetrator’s identity				Diversity * Identity							
	Only one type (*n* = 114)		More than one type (*n* = 103)		Stranger (*n* = 24)		Acquaintance (*n* = 193)		One * Stranger (*n* = 15)		More than one* Stranger (*n* = 9)		One * Acquaintance (*n* = 99)		More than one * Acquaintance (*n* = 94)	
Variables	*M*	*S.D.*	*M*	*S.D.*	*M*	*S.D.*	*M*	*S.D.*	*M*	*S.D.*	*M*	*S.D.*	*M*	*S.D.*	*M*	*S.D.*
Anger	6.81	4.06	6.05	3.86	7.25	4.81	6.35	3.86	6.33	3.85	8.78	6.04	6.88	4.11	5.79	3.52
PTSD symptoms	6.99	4.10	7.23	4.42	7.08	4.63	7.11	4.25	5.67	2.85	9.44	6.13	7.19	4.24	7.02	4.20
Depressive symptoms	4.73	3.06	4.65	3.05	5.21	2.90	4.63	3.07	5.27	2.28	5.11	3.89	4.65	3.16	4.61	2.98
*F* (3, 207) (η^2^)	2.68* (.04)				.82 (.01)				3.46* (.05)							
					Stepdown analyses											
Anger *F* (3, 207) (η^2^)	.60 (.00)				1.94 (.01)				4.06* (.02)							
PTSD symptoms *F* (4, 206) (η^2^)	3.36 (.02)				.27 (.00)				1.10 (.01)							
Depressive symptoms *F* (5, 205) (η^2^)	4.01* (.02)				.27 (.00)				5.10* (.02)							

*M* mean, *S.D.* standard deviation, + plus, *η*^*2*^ partial eta squared.

* *p* < .05; ** *p* < .01

The effects observed for depressive symptoms are not interpreted. Given the small number of subjects in certain groups and the absence of significant results for these symptoms in the preceding analyses, proceeding this way was deemed more conservative

A last 2 (diversity of the manifestations) × 2 (presence or absence of injuries) factorial MANOVA was performed for the 217 young victims, always with the same dependent variables. The results are non-significant and are not presented in tables.

## Discussion

This study aimed first at assessing the differences between youth having experienced physical community violence, compared with those not having had such an experience, on the following psychological variables: anger, PTS symptoms, and depressive symptoms. The results partially confirm the hypothesis that youth having experienced violence would present more psychological problems, since this finding was observed only for anger. It is important to emphasize that the variance shared between anger, PTS, and depression in our sample contributed to a significant result being observed for anger only, given the analysis strategy selected. In this context, the measure of anger could be interpreted as an indicator of a more general psychological ill-being in the adolescents from the general population.

This finding is consistent with those found in the literature, underscoring a strong association between community violence and aggressiveness (Fowler et al. 2009). For instance, being a victim or witness of community violence is strongly associated with the use of aggressive behaviors by youth (Scarpa 2001). An explanation is that violence exposure contributes to the development of hostile attributions towards the actions of others and to the normalization of aggressive behaviors (Janosz et al. 2008). Furthermore, youth exposed to violence have limited opportunities to interact appropriately with their peers and to develop positive socializing experiences. Thus, they learn to adopt the same violent behaviors as those that they themselves have suffered or witnessed (Ostrov 2010). Despite a small effect size, the current study shows that it is possible to detect an association between exposure to physical community violence and psychological outcomes in adolescents from the general population. Since various forms of interpersonal violence remain relatively infrequent and mild in the general population, small effect sizes are expected and the capacity of detecting them is a sound result in itself.

Findings also support the hypothesis that sex does not interact with exposure to community violence to explain differences in adolescents’ psychological symptoms. First, there was no main effect of sex on the level of anger, PTS symptoms, and depressive symptoms reported by adolescents. Nonetheless, the current literature maintains that boys are more likely than girls to report aggressive and antisocial behaviors (Afifi et al. 2011). In this regard, it is important to keep in mind that the measure used in the present study assesses the emotional and cognitive experience of anger (feeling furious, feeling cross, wanting to scream) rather than aggressive behavior per se. It is possible that boys and girls feel anger in a similar manner but that they express it differently. Second, no interaction effect between sex and physical community violence experience contributed to explain differences in the level of psychological outcomes. This is consistent with the McDonald and Richmond’s review: being a boy or a girl makes no difference in the level of psychological problems associated with physical community violence. Even though adolescent girls are generally less exposed to physical community violence than their male counterparts (Dubé et al. 2014; Rose and Rudolph 2006), those who are exposed report the same level of difficulties than boys.

Next, this study aimed to evaluate whether the diversity of the reported manifestations of physical community violence, as well as being a direct victim or a witness, resulted in differences in the three psychological variables of interest among the 435 youth who reported experiencing at least one manifestation of physical community violence. The results support the hypothesis that direct victims present slightly more anger than witnesses. One possible explanation is based on the theoretical conceptualization of interpersonal violence among adolescents proposed by Crowther et al. (2013). These authors underscore the aspect of identity that is predominant during this period in life and that could bring youth who are victims of violence to resort to violence themselves as an adaptation strategy. This strategy would promote their identity as a strong, brave and threatening person in the eyes of others, allowing them to counter the violence that they have suffered and to prevent future violence. However, it seems that this strategy does not help reduce the risks of sustaining violence, but rather increases it (Stewart et al. 2006). Furthermore, the use of such behaviors may lead to a vicious circle where the more youth resort to violence, the more they feel angry and the more this anger leads to the use of violence (Ostrov 2010; Scarpa and Ollendick 2003). Because witnesses do not have to defend their own identity, since they are not directly involved in the violent event, they may be less inclined to use violent behaviors or to report feelings of anger associated with the events in question.

Meanwhile, significant results were found for diversity of the reported manifestations of physical community violence, but only in the group of direct victims of violence, and in interaction with being acquainted with the perpetrator. Findings show that youth victimized by a stranger express significantly more anger than those victimized by an acquaintance, but only in cases where they reported sustaining a diversity of manifestations of violence (interaction effect). However, this result must be interpreted with caution, because only a small number of respondents (*n* = 24) reported being victims of strangers only, and only nine had suffered several manifestations of violence on the part of strangers.

In sum, the highest level of anger is reported by the victims of several manifestations of physical violence perpetrated by strangers, whereas the lowest level of anger is observed in victims of several manifestations of physical violence by acquaintances. Considering that the perpetrator who is an acquaintance is generally a youth under 18 years old, such as a friend, a peer, or a neighbor (Dubé et al. 2014), this special relationship may play a role in the expression of anger. Friends take up a lot of room in young people’s lives during adolescence, a period where creating numerous significant relationships with peers and being accepted by them is a major concern (Waldrip et al. 2008). It is thus possible that youth who have been victimized several times by acquaintances will prefer to remain impassive and to not retaliate against the violent acts experienced, to avoid rejection by their peers. The victims could come to deny the feelings experienced or to avoid thinking about them, to preserve the relationship with their peers and their sense of belonging.

Finally, the presence of injuries does not help explain the psychological difficulties of the young victims in this study. The literature indicates that being physically injured during a violent event influences the psychological symptoms reported, especially of PTS (Kennedy and Ceballo 2014; Ozer et al. 2008). Perceiving that one’s life is in danger and sustaining several physical injuries increase the risk of presenting such symptoms (Martin et al. 2006). The absence of significant results in this study is probably due to the very small number of youth reporting this type of experience. Among the 77 youth who reported being physically injured during an incident of community violence, only four reported serious injuries, such as broken bones or concussions. Others reported mostly superficial injuries, such as bruises or cuts. Sustaining superficial injuries may not be sufficient to develop PTS symptoms.

## Strengths and Limitations of the Study

This study has strengths as well as limitations. The use of a large, randomly recruited populational sample favors the generalization of the results and helps further understand all aspects of the adolescents’ community violence experience. Since the adolescents were interviewed directly, their opinion on this reality is presented in this study. Considering the distinction between being a direct victim of community violence and being only a witness helps bring out the subtleties of the youth’s experience of violence. Additionally, taking several variables into account to describe the youth’s experience of physical community violence more specifically, such as being acquainted or not with the perpetrator and the presence or absence of injuries, helps to understand which parameters make community violence harmful for adolescents in the general population.

Speaking to the limitations of the study, the use of only three psychological health indicators precludes an in-depth assessment of the psychological state of the youth in the sample as it relates to their community violence experience. Other symptoms normally associated with community violence, such as anxiety, aggressive behaviors and low self-esteem, would have helped provide a more complete picture. The use of a measure of community violence derived from an instrument not designed for this purpose (the JVC) also constitutes a limitation to this study. Finally, the use of a cross-sectional design precludes the conclusion of causal relationship between community violence and psychological health.

## Conclusion

Given current concerns regarding youth’s mental health, the main contribution of this study is to show that experiences of physical community violence could contribute to disturbing girls and boys alike, notably by feeding feelings of anger. Other studies are needed to establish the direction of this relationship. Even if the effects observed are small, the fact that they were detected in the general population is not benign and makes these results original, since most previous studies have been conducted with high-risk samples. The study confirms that being a direct victim is associated with more anger than being exposed as a witness. It also suggests that being victimized by an acquaintance (most of the time a peer) may have a suppressor effect on this anger, since the youth may want to preserve the relationship. However, given the limitations of the sample, this last result must be interpreted with caution and will need to be replicated.

## Footnotes

1 The correlation matrices are not presented here for the sake of brevity. However, they are available from the corresponding author.
